# Evaluating the accuracy of a functional SNP annotation system

**DOI:** 10.1186/1471-2105-10-S9-S11

**Published:** 2009-09-17

**Authors:** Terry H Shen, Christopher S Carlson, Peter Tarczy-Hornoch

**Affiliations:** 1 Departments of Biomedical & Health Informatics, University of Washington, Seattle, WA, USA; 2 Genome Sciences, University of Washington, Seattle, WA, USA; 3 Computer Science and Engineering, University of Washington, Seattle, WA, USA; 4 Pediatrics, University of Washington, Seattle, WA, USA; 5 Fred Hutchinson Cancer Research Center, Seattle, WA, USA

## Abstract

Many common and chronic diseases are influenced at some level by genetic variation. Research done in population genetics, specifically in the area of single nucleotide polymorphisms (SNPs) is critical to understanding human genetic variation. A key element in assessing role of a given SNP is determining if the variation is likely to result in change in function. The SNP Integration Tool (SNPit) is a comprehensive tool that integrates diverse, existing predictors of SNP functionality, providing the user with information for improved association study analysis. To evaluate the SNPit system, we developed an alternative gold standard to measure accuracy using sensitivity and specificity. The results of our evaluation demonstrated that our alternative gold standard produced encouraging results.

## Introduction

The mission of population health studies is to promote the public's health and prevent diseases. Nine of the top ten leading causes of mortality in the US have significant genetic components [1]. To illuminate the genetic elements of common human disease, researchers must thus study genetic variation.

Single nucleotide polymorphisms (SNPs) are a powerful tool used in association studies looking at genetic variation. Differences in the genome occur most frequently through SNPs; it has been estimated that between 5 and 15 million common SNPs exist, depending on the population studied [2]. Due to the fact that SNPs have a lower mutation rate than microsatellites, are easier to genotype in an automated fashion, and occur at much greater density in the genome, SNP markers are being used increasingly in association studies. Although millions of SNPs exist, the vast majority of these polymorphisms are not functional, so the identification of functional polymorphisms are one of the trickiest challenges faced by population geneticists and molecular biologists; regardless of the location of the SNP in question (coding vs. non-coding, genic vs. non-genic), prediction of function is difficult.

Creating a resource to integrate knowledge of polymorphic positions in the genome with functional predictions will facilitate the identification of causal polymorphisms. Current databases and prediction algorithms can provide annotated information ranging from protein function to transcription factor binding to evolutionary conservation. However, none of these resources attempts to consolidate information from diverse data sources, or attempts to use inference rules to synthesize new knowledge from the existing information. Our system, SNP Integration Tool (SNPit), provides such a tool [3].

## Related work

There are a limited number of resources out there that integrate SNP resources, an even smaller number of which have had evaluations of their performance. The UCSC Genome Browser [4] is one of the primary resources used to look at integrated information about SNPs. The underlying architecture is not a general purpose data integration system. In fact, none of the other systems use a general purpose data integration framework as their core. There are only a few data integration tools that have been developed to select SNPs based on functional properties. PupaSNPFinder and PromoLign take a gene-centric approach and provide information on the transcriptional effects of SNPs [5,6], with PupaSNPFinder looking at the validation status of SNPs, but not its functional role. SNPer focuses on data export tools, the application provides annotation information, but a limitation is that it does not focus on functional prediction, the resource compares itself with other SNP databases as its evaluation technique [7]. SNPselector provides a web selection program that looks at SNP properties, including the concept of linkage disequilibrium and prioritization, it uses a case study approach and and thus only looks at the results of seven hundred SNPs [8] (among potentially millions).

In all these systems, only subsets of all possible SNP predictors are examined and none of the systems evaluate their performance regarding the functional annotation of SNPs. In addition, all of these annotation systems use a local data warehouse approach to storing data, limiting both its extensibility and accuracy in terms of up-to-date information. Finally, though there are numerous databases available that catalogue specific parameters of genetic variation data, there is no central way to query a region for SNPs with strong functional predictions based on data from multiple tools. Using a generalized federated database system approach, SNPit addresses these limitations making it easier to add new sources and adapt to evolving sources. SNPit can thus easily integrate diverse structural, evolutionary, and expression data for SNPs, and can be extended to other data types as they become available. Using SNPit, population geneticists will be able to more efficiently identify candidate causative genomic variations for human disease after an initial statistical association has been found.

## Methods

### SNPit integration tool

SNPit is built on the BioMediator foundation [9]. BioMediator is a federated data integration system: meaning that the owners of the data retain their ownership [10]. Federated data integration systems have an advantage over traditional data warehousing techniques for a variety of reasons including: the ability to retrieve up to date information without having to refer back to the original data sources, maintaining a manageable computing environment since a cumbersome database is not necessary for storage, and allowing for flexibility in querying of databases, since users and developers are not bound by the data format of the various sources. The user has the ability to traverse over multiple databases, and query only the information pertinent to the question being asked. The user may also integrate private data sources, allowing for integration of proprietary information in analysis.

Currently, the SNPit integration system has been implemented across several biological domains, linking data related to functional SNPs from sources accessible through BioMediator. BioMediator has interfaces to over 15 public databases (such as Entrez, Swissprot, and OMIM) as well as many private databases of experimental results (including phenotypic databases, genetic databases, imaging databases, and expression array databases) [11].

**Figure 1:**
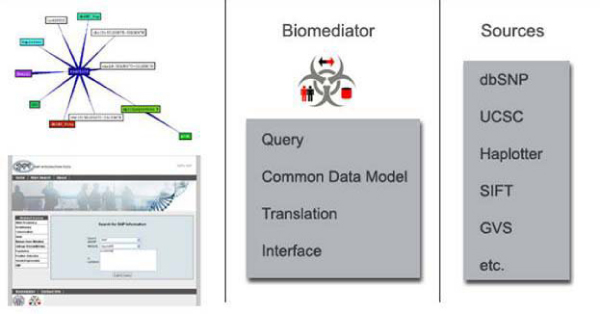
**Diagram of SNPit components**.

The central element to BioMediator's generalizability is the source knowledge base (SKB). The SKB includes descriptions of the data sources, mappings from the source to the mediated schema, and the mediated schema itself. The mediated schema is a general outline that incorporates all the common objects and mappings for the data sources. The SKB can be customized for the various end users. The other three components to the system include: 1) generalized wrappers that translate the data sources syntactically; 2) a metawrapper that goes between the data source and the user query and translates semantically; and 3) the query processor which allows users to query against the mediated schema (Figure 1).

### Evaluation

The challenge of evaluating SNP annotation is that there is no true gold standard, particularly for complex diseases where the genomic component does not explain the environmental influence, both of which are necessary for a change in phenotype. To address this challenge, we focused on monogenetic diseases for the evaluation component, using HGMD which provides published evidence on annotation of Mendelian genes. We examined three different annotation categories: a) synonymous/non-synonymous, b) splicing, and c) regulatory SNPs. Synonymous/nonsynonymous SNPs occur in the coding region of the genome and affect whether or not the amino acid is changed (non-synonymous being a change in amino acid). Splicing SNPs occur at the intron/exon boundary when proteins are being made, a SNP at this location can affect how the intron is spliced out. Regulatory SNPs occur in the promoter of the sequence and are thought to affect gene expression.

For example, to evaluate the missense/nonsense category of a particular gene, we retrieved the mutations from HGMD, performed some manipulations so that were properly formatted for SIFT analysis, and then found the corresponding conditional probabilities that predict whether the change in amino acid detrimentally affects the protein. For the probabilities that are low, that would suggest that a change in amino acid would be detrimental. We then chose a threshold (0.2) to determine which SNPs would be scored as a true positive. For the true positives category, we found the unique sequences of those SNPs that are in the same candidate gene as before, and found the corresponding sequences for those rs numbers and submitted them to SNPit's protein function prediction section. The same threshold was selected for determining which SNPs would be deleterious.

Using this alternative ("pseudo") gold standard, we measured accuracy initially using sensitivity and specificity of the SNPit system. We submitted the same annotation category to both SNPit and HGMD and measuring the difference between the two. We repeated the process for five different genes (CFTR, BRCA1, tp53, NF1, BRCA2). Each gene contained around 700–1,500 SNPs. Each SNP was submitted to both systems.

The alternative gold standard uses HGMD as its main source of positive evidence for a functional SNP in one of the three categories and dbSNP as its source for non-evidence (thus a SNP in dbSNP with no corresponding HGMD entry is presumed to be lack of evidence for function). We defined sensitivity as the ability to detect the positive evidence for SNP functionality, and specificity as the ability to detect non-evidence for SNP functionality. A true positive (TP) result would be when the test is correctly classified as functionally relevant, a true negative (TN) result would be when the SNP is correctly classified as being non-functional; false positive (FP) would be when the test is incorrectly classified as being functional, and false negative (FN) would be when the test incorrectly classifies the test as being nonfunctional [12]. HGMD provided the information for the true positives and false negatives. SNPs found in dbSNP but not found in HGMD, provided the information for the false positives and true negatives. Non-synonymous SNPs were tested again SNPit's SIFT category, regulatory SNPs were tested against SNPit's TFSearch category, and splice site SNPs were tested against SNPit's BDGP category.

In addition, we calculated the sensitivity and specificity of the entire federated system. Rather than separating the SNPs being tested by category as done during the first phase of the evaluation, we tested how the entire federated system returns annotation information on tested SNPs. Thus, in the second phase of the evaluation, we examined whether or not any of the SNPs are flagged by any of the categories as functionally promising. We tested HGMD against all three categories: non-synonymous, regulatory, and splicing, with true positives defined as those that are flagged as interesting by at least one of the three categories. Likewise, we ran those SNPs that are in dbSNP but not in HGMD against all three categories to establish the false negatives.

## Results

**Figure 2:**
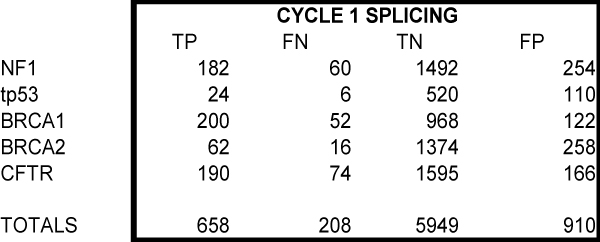
**Subset of results for cycle 1 splicing category**.

**Figure 3:**
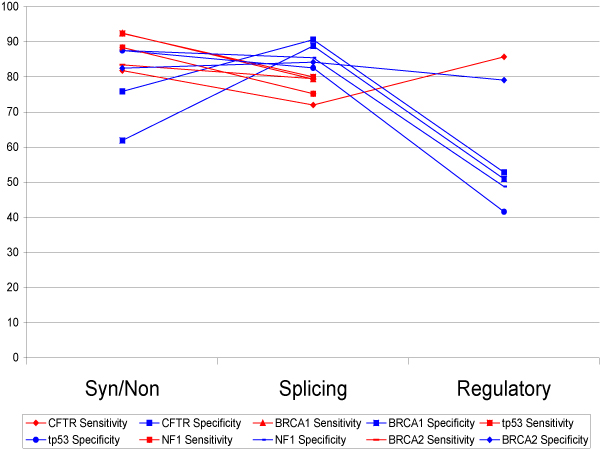
**SNP evaluation for cycle 1**.

We evaluated five candidate genes: CFTR, BRCA1, tp53, NF1, BRCA2 using both evaluation techniques as described in the Methods section. A portion of the SNPs evaluated in cycle 1 for the splicing category is presented in Figure 2. The sensitivity and specificity for all the candidate genes in the annotation categories synonymous/nonsynonymous and splicing fall around 80% (Figure 3). The sensitivity and specificity measures for the regulatory category were lower, demonstrating perhaps the difficulty in prediction of regulatory mutations.

**Figure 4:**
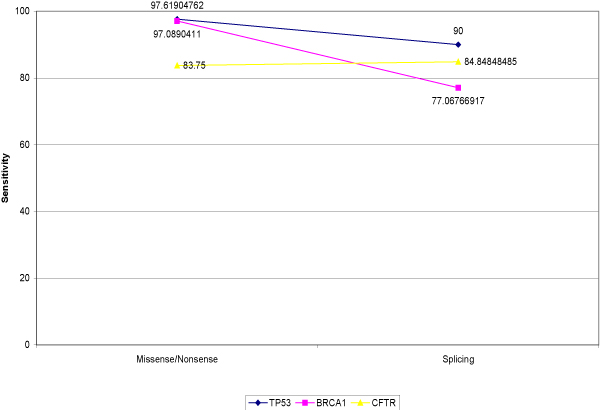
**SNP evaluation for cycle 2**.

For the second cycle, in which we combined all the categories together, the results improved (Figure 4). For the tp53 gene, the sensitivity was 97.69% for the missense/nonsense category and 90% for the splicing category. For the BRCA1 gene, the sensitivity was 97.09% for the missense/nonsense category and 70.07% for the splicing category. For the CFTR gene, the sensitivity was 83.75% for the missense/nonsense category and 84.85% for the splicing category.

**Figure 5:**
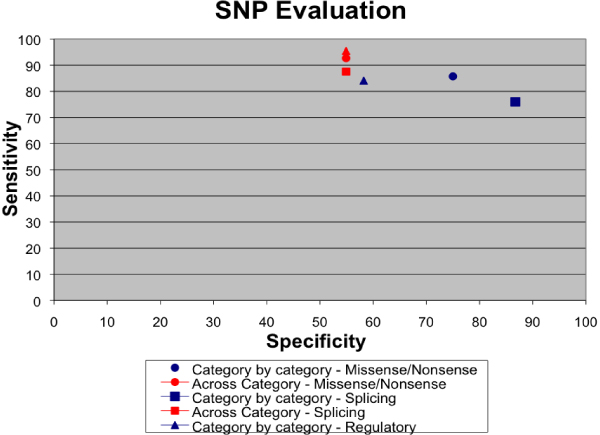
**Sensitivity versus specificity graph for both cycles**.

The graph of sensitivity versus specificity for both cycles demonstrated that when you group the categories together, an increase in sensitivity is observed (Figure 5). This increase is due to the combination of all three categories in cycle 2. For example, those SNPs that were originally in the regulatory category yielded a positive prediction for the splicing category. Given an overall false positive rate of around 1 in 7 for splice predictions (910 out of 6,859 in Cycle 1, see Figure 2), this suggested that a portion of regulatory SNPs were actually acting through a splicing mechanism.

## Discussion

Informatics has opened up the field of genomic research, providing new computational approaches to gathering and analyzing genetic data, both of which can provide more powerful tools to determining genetic influences of diseases. The number of SNP annotation systems that are currently available is limited, and noneof them to our knowledge have had evaluated the functional annotation accuracy of their system. We have built and tested a system (SNPit) that addresses gaps in the existing systems with regards to SNP functional annotation. In addition, in order to evaluate the SNPit system we created an alternative gold standard to measured accuracy using sensitivity and specificity. The initial results are encouraging, though improvements are certainly still possible.

Our team continues to improve and expand upon SNPit through the elicitation of user feedback. The SNPit system is currently accessible over the Internet to both population geneticists and molecular biologists. Addition of a logical inference engine (Jess) on top of the BioMediator data integration platform has been shown to successfully predict the functional annotation of anonymous sequences [13].

Furthermore, recent work had demonstrated that support for probabilistic inference on top of the BioMediator data integration system can improve protein functional annotation [14]. Thus, we are working to create Jess inference rules and uncertainty measures to build expert knowledge into the SNPit tool and incorporate uncertainty measures into the system. Our goal is to create a system that is easy to use and thoroughly comprehensive for the purpose of functional SNP annotation.

## Conclusion

The future of both public health and translational informatics lies in the concept of preventive, predictive, and personalized medicine. The discoveries from genetic variation studies can contribute to preventive, predictive, and personalized medical discoveries through a variety of methods including the discovery of new drug targets andearlier identification of subsets of patients for surveillance purposes.

Once a genome wide association study is completed, researchers need to understand the biological mechanism between the observed phenotype and the genetic variation, identifying the annotation of the SNPs being studied is an important component. Genetic researchers faced with a long list of SNPs associated statistically with a phenotype need to be able to narrow this list focusing on those SNPs most likely to be causally related to the phenotype. Our system, SNPit, provides such an annotation tool. We define the users of our proposed tool, SNPit, to be genetic epidemiologists, public health geneticists, biological scientists, molecular biologists, and any other scientists that work with genome wide association studies and SNPs.

## Availability and requirements

Project name: SNPit

SNPit homepage: 

Recommended internet browsers: Firefox or Internet Explorer

Programming language: Java

## Competing interests

The authors declare that they have no competing interests.

## Authors' contributions

TS designed, implemented, and evaluated the SNPit system and wrote the primary draftsof this paper. CC provided expertise on SNP annotation and guided the development of the project. PTH provided biomedical informatics expertise and guided the development of the project. All authors participated in the drafting of this paper and approved the final draft for submission.
